# Vision-Language Model for Generating Textual Descriptions From Clinical Images: Model Development and Validation Study

**DOI:** 10.2196/32690

**Published:** 2024-02-08

**Authors:** Jia Ji, Yongshuai Hou, Xinyu Chen, Youcheng Pan, Yang Xiang

**Affiliations:** 1 Shenzhen Institute of Information Technology Shenzhen China; 2 Peng Cheng Laboratory Shenzhen China; 3 Harbin Institute of Technology Shenzhen China

**Keywords:** clinical image, radiology report generation, vision-language model, multistage fine-tuning, prior knowledge

## Abstract

**Background:**

The automatic generation of radiology reports, which seeks to create a free-text description from a clinical radiograph, is emerging as a pivotal intersection between clinical medicine and artificial intelligence. Leveraging natural language processing technologies can accelerate report creation, enhancing health care quality and standardization. However, most existing studies have not yet fully tapped into the combined potential of advanced language and vision models.

**Objective:**

The purpose of this study was to explore the integration of pretrained vision-language models into radiology report generation. This would enable the vision-language model to automatically convert clinical images into high-quality textual reports.

**Methods:**

In our research, we introduced a radiology report generation model named ClinicalBLIP, building upon the foundational InstructBLIP model and refining it using clinical image-to-text data sets. A multistage fine-tuning approach via low-rank adaptation was proposed to deepen the semantic comprehension of the visual encoder and the large language model for clinical imagery. Furthermore, prior knowledge was integrated through prompt learning to enhance the precision of the reports generated. Experiments were conducted on both the IU X-RAY and MIMIC-CXR data sets, with ClinicalBLIP compared to several leading methods.

**Results:**

Experimental results revealed that ClinicalBLIP obtained superior scores of 0.570/0.365 and 0.534/0.313 on the IU X-RAY/MIMIC-CXR test sets for the Metric for Evaluation of Translation with Explicit Ordering (METEOR) and the Recall-Oriented Understudy for Gisting Evaluation (ROUGE) evaluations, respectively. This performance notably surpasses that of existing state-of-the-art methods. Further evaluations confirmed the effectiveness of the multistage fine-tuning and the integration of prior information, leading to substantial improvements.

**Conclusions:**

The proposed ClinicalBLIP model demonstrated robustness and effectiveness in enhancing clinical radiology report generation, suggesting significant promise for real-world clinical applications.

## Introduction

Radiology reports offer essential textual descriptions of radiographs and play a pivotal role in the medical diagnosis and treatment process. Their precise interpretation can directly influence patient outcomes, underscoring the gravity of each assessment. However, even for seasoned radiologists, interpreting these images can be a meticulous task, often taking several minutes per image. In an era where timely medical intervention can be lifesaving, streamlining this process becomes imperative. Recognizing the immense potential to ease the workload of the health care sector and improve patient care, there has been a growing interest in the research for automatic radiology report generation.

**Figure 1:**
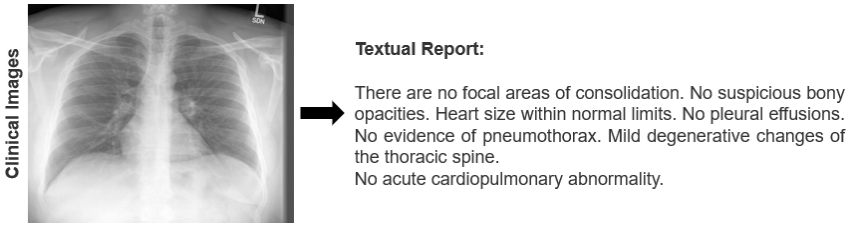
Example of a radiology report generation task.

As shown in Figure 1, several attempts have been made in the medical field to create medical reports from images. In the early stage, most researchers used traditional deep learning methods, such as convolutional neural networks (CNNs) and recurrent neural networks (RNNs), to produce radiology reports. IU X-RAY proposed by Demner-Fushman et al [1] was a significant step in this direction. In addition, Shin et al [2] innovatively applied a CNN-RNN model for structured report creation. Wang et al [3] used a nonhierarchical CNN-long-short term memory approach, emphasizing both semantic and visual cues. Vinyals et al [4] introduced visual attention mechanisms in the realm of image captioning with CNN-RNN structures. Subsequently, radiology report creation has evolved to adopt transformer-based models [5,6]. The Knowledge-Driven Encode, Retrieve, Paraphrase method was proposed by Li et al [7] to ensure accurate medical report generation. To better recognize common radiographic findings, Yuan et al [8] suggested pretraining encoders with an array of chest x-ray images. Chen et al [9] put forward the idea of producing radiology reports using a memory-centric transformer. Meanwhile, Pino et al [10] advocated for a template-driven methodology for x-ray report generation. In their model, clinical templates are defined for each abnormality, signaling its presence or lack thereof. However, this method falls short in conveying specific patient details like anatomical positions or size dimensions. Addressing this, Wang et al [11] introduced a template-oriented multiattention report generation model, which is tailored especially for standard reports.

Recently, vision-language models (VLMs) [12-15] have become leading approaches, which use pretrained transformer models to handle both visual and textual data at the same time. These models are very good at understanding and creating content based on images and texts. One key feature is cross-modal learning [16,17], where VLMs learn to match specific image patterns with their related descriptions or findings. This understanding helps in making reports that are more relevant and accurate. VLMs have the potential to greatly improve radiology report generation by increasing accuracy, making processes faster, and ensuring consistency. However, it is important to address challenges related to data quality, integration, and rules when using VLMs in clinical settings. Thus, designing an effective fine-tuning method to boost VLM’s knowledge and understanding of medical images and texts is a very interesting research direction.

In this study, we fine-tune a medical VLM named ClinicalBLIP through a multistage fine-tuning strategy for the radiology report generation task. First, a joint optimization method that combines self-attention fine-tuning via low-rank adaptation (LoRA) [18] with layer normalization [19] is proposed to enhance the understanding of clinical images by a general visual encoder. The training target is the text generation loss of the large language model (LLM) without introducing extra clinical image-text pairs for further pretraining. Second, the joint fine-tuning process for both the image-text transformation layer and the multilayer perceptron (MLP) layer of the language model is designed to allow the LLM to draw upon its internal capability to generate the final report. In addition, we further incorporate the prior information to light the specialized clinical knowledge inherent in the LLM. Also, the clinical tag and brief description of the image as a text prompt are fed into the model for training and prediction. Experiments were conducted on the IU X-RAY [1] and MIMIC-CXR [20] data sets. We compared the proposed model with 11 competitor methods and analyzed the performance in several aspects. It is demonstrated that the proposed ClinicalBLIP achieved state-of-the-art performance and can effectively combine the introduced textual prior knowledge with clinical images to generate better reports.

## Methods

### Data Set

We evaluated our proposed method on the IU X-RAY [1] and MIMIC-CXR [20] data sets. Both data sets have been automatically deidentified.

The IU X-ray data set comprises 7470 images and 3955 reports. The images consist of chest x-rays originating from Indiana University. Each report in the data set primarily encompasses multiple attributes such as comparison, indications, findings, and impressions. Reports with empty findings were excluded, resulting in 3337 remaining reports. Subsequently, we divided the remaining reports into training and testing sets in a 4:1 ratio, yielding 2668 reports for training and 669 reports for testing.

The MIMIC-CXR data set was created by the Massachusetts Institute of Technology. The images are sourced from 65,379 patients who presented to the Beth Israel Deaconess Medical Center Emergency Department between 2011 and 2016. We used 152,173 medical reports for training and 1196 reports for testing. In this data set, each data entry comprises a specific report and 1 to 3 corresponding images.

### Overview of the Proposed Method

**Figure 2:**
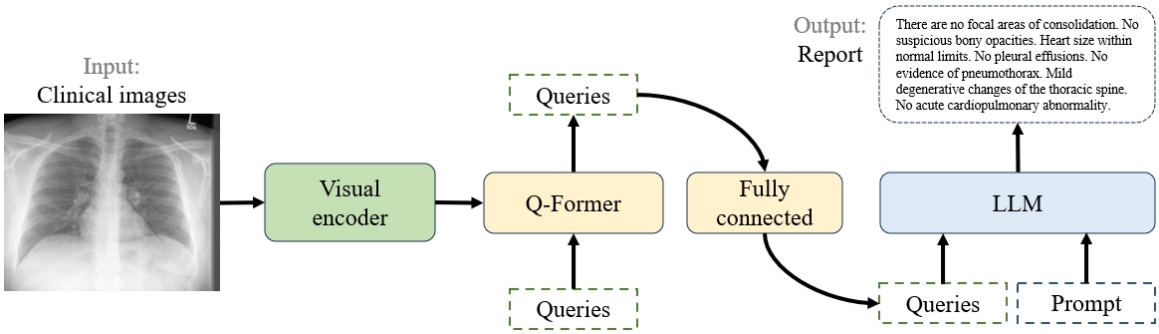
Overview of the proposed ClinicalBLIP model. LLM: large language model; Q-Former: query transformer.

Our work aims to transform clinical radiographs, accompanied by additional information, into textual descriptions that convey the same semantic meaning as the images. To achieve this, we introduce the ClinicalBLIP model, as depicted in Figure 2. This model comprises three core modules: (1) a visual encoder for converting clinical images into semantic representations; (2) query transformer (Q-Former), a crucial component for bridging the image-text gap; and (3) a LLM for generating textual reports based on queries learned from Q-Former and textual prompts.

Initially, we briefly introduce the structure and pretraining of the ClinicalBLIP, which draws inspiration from Li et al [21], especially how Q-Former as an intermediate module effectively connects visual and textual data. Subsequently, we delve into the details of how to effectively fine-tune the task of radiology report generation.

### Q-Former to Bridge the Modality Gap

**Figure 3:**
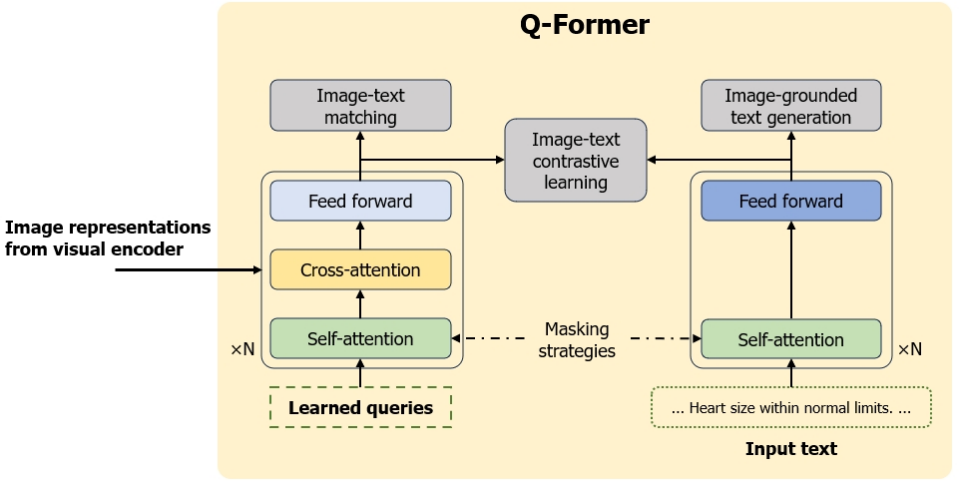
Model architecture of query transformer (Q-Former).

Q-Former is designed to link a fixed image encoder with a standard LLM. Notably, it can extract a consistent set of features from the visual encoder, regardless of the input image resolution. As shown in Figure 3, the model is composed of two primary transformer submodules: (1) an image transformer for direct interaction with the visual encoder and (2) a text transformer that serves as both encoder and decoder. The efficacy of the Q-Former is greatly influenced by learnable query embeddings, which facilitate self-attention and cross-attention layer interactions. These embeddings also enable communication with text through similar attention mechanisms. During its 2 pretraining phases, that is, vision-language representation learning and vision-to-language generative learning, Q-Former uses distinct attention masks for specific tasks, controlling the interaction between queries and text.

### Vision-Language Representation Learning From Visual Encoder

In the representation learning phase, Q-Former, connected to a frozen visual encoder, undergoes pretraining with image-text pairs. The objective here is to train the model to enable queries to extract visual representations corresponding to the text. Inspired by Li et al [22], 3 pretraining tasks are jointly optimized, using the same input format and model parameters. As illustrated in Figure 3, these tasks include image-text contrastive learning, image-grounded text generation, and image-text matching. Image-text contrastive learning aligns image and text representations by contrasting the similarity of a positive image-text pair against that of negative pairs. Image-grounded text generation encourages the Q-Former to compel the queries to extract visual features that contain the whole information of the text. Image-text matching seeks to capture fine-grained alignment between image and text representations through a binary classification task. Each task uses a specific attention-masking strategy to control the interaction between queries and text.

### Vision-to-Language Generative Learning From LLM

During the generative pretraining phase, Q-Former, connected to a frozen LLM, leverages its language generation capabilities. A fully connected layer is used to linearly project the output query embeddings to match the dimension of the LLM’s text embedding. These embeddings then act as visual prompts, guiding the LLM based on the visual representation captured by Q-Former. Since Q-Former has been trained to extract visual representations that are informative for language, it effectively serves as an information filter, providing only the most relevant information to the LLM and excluding unnecessary visual details. This setup reduces the load on the LLM to learn vision-language alignment, mitigating the risk of the catastrophic forgetting problem.

### General Vision-Language Instruction Tuning

Following the pretraining phases, as in Dai et al [23], Q-Former undergoes a vision-language instruction tuning process. Here, the LLM integrates visual encodings from Q-Former with additional instruction text tokens. The instruction interacts with the query embeddings through the Q-Former’s self-attention layers. This interaction aids in extracting relevant image features, which are further provided to optimize the LLM for following instructions. Both quantitative and qualitative analyses confirm the effectiveness of the instruction tuning process in achieving vision-language zero-shot generalization.

### Effective Fine-Tuning of Radiology Report Generation

**Figure 4:**
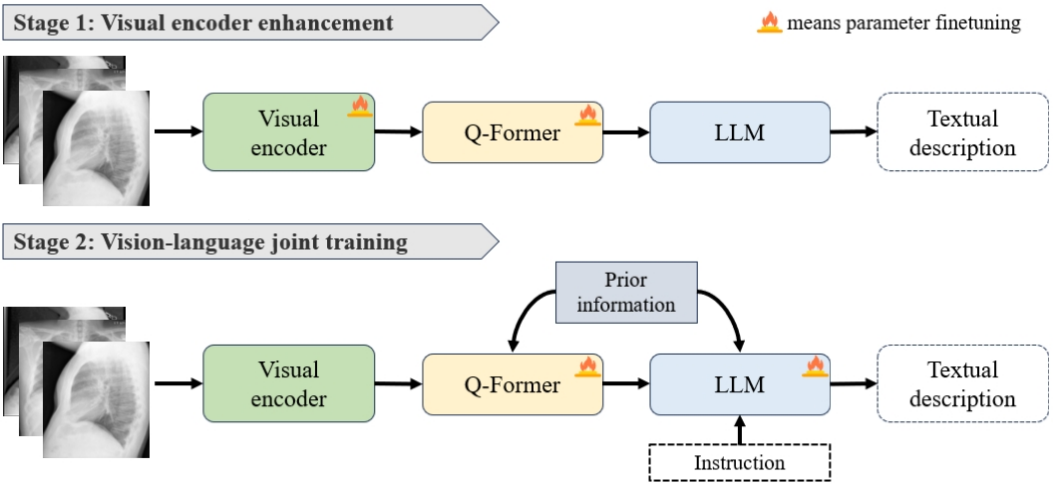
Multistage fine-tuing on radiology report generation. LLM: large language model; Q-Former: query transformer.

To enhance the performance of a general visual encoder and an LLM for medical image understanding and report generation, various aspects need careful consideration. As shown in Figure 4, a multistage parameter fine-tuning approach is used to improve model performance, namely visual encoder enhancement and vision-language joint training.

In the first stage, the model’s weights are adjusted to focus more on relevant features within medical images. This refinement aids in understanding critical elements such as lesions, organs, and more. Concurrently, layer normalization is applied to maintain a consistent response across varying image scales and brightness levels. The primary objective here is the generation loss of the LLM, aiming to improve the quality of final reports by enhancing the visual encoder’s ability to use visual information more effectively during report generation, without the need for additional medical text data for further pretraining.

In the second stage, the joint training process encompasses the fusion of visual and textual inputs, and crucially, the incorporation of the attention layer and MLP layer of the LLM. The model simultaneously processes information from the visual encoder and textual sources. The attention layer enables dynamic focus on specific regions of medical images, aligning with features crucial for report generation. Meanwhile, the MLP layer transforms the combined visual-textual data, boosting the model’s ability to generate contextually accurate and coherent medical reports. The whole approach ensures full use of the model’s attention and transformation capabilities, yielding medically precise and linguistically sound reports, thus effectively bridging the gap between visual and textual data.

Moreover, general LLMs often struggle with the absence of specialized medical domain knowledge in the medical report generation task. To mitigate the issue, we incorporate the prior information into the model during the second stage. Specifically, medical tags related to the medical image and a brief image description are embedded as text prompts. In training, these prompts are linked to corresponding medical images, facilitating the model’s comprehension of the image content. This association enables the model to better learn medical domain-specific terms and concepts. This embedding of text prompts guides the model with domain knowledge, addressing its limitations in the medical field. During prediction, these prompts provide additional contextual information, enabling the model to better comprehend medical images, identify features within them, and express the medical reports in a more professional manner.

In our research, we analyze 2 relevant data sets: IU X-RAY [1] and the MIMIC-CXR [20]. Each of them has unique prior information as input. The IU X-RAY data set enriches the model with essential prior information, including “problem,” “image,” and “indication.” The input template for the IU X-RAY data set is formatted as follows:

“Problems: {problem} \n Image: {image} \n Indication: {indication} \n”,

exemplified by “Problems: normal \n Image: Chest, 2 views, frontal and lateral \n Indication: Pruritic \n”.

In contrast, the MIMIC-CXR data set lacks direct access to similar prior information. To maintain consistency, we use the CheXBERT [24] model to extract medical observations from the reports within the MIMIC-CXR data set. The input template for this data set is formatted as follows:

“Symptoms of existence: {} \n Symptoms of non-existence: {} \n”,

illustrated by “Symptoms of existence: Cardiomegaly, Atelectasis \n Symptoms of non-existence: Edema, Consolidation \n”.

### Experimental Settings

We adopt the InstructBLIP [23] as the base model, in which contrastive language-image pretraining [13] and Flan-T5-XL [25] are used as visual encoders and LLM structures, respectively. In the training phase, we integrated LoRA [18] into both the visual encoder and the language model. This integration of LoRA was strategically implemented within the query projection and value projection stages during self-attention operations, enhancing the model’s ability to capture and leverage relational information. For the training process, we configured our settings as follows: a batch size of 3 was used, and gradient accumulation was carried out over 4 steps to facilitate stable and efficient training. The initial learning rate for the Q-Former parameters was set to 1×10^–4^, while the initial learning rate for the LoRA-related parameters was established at 5×10^–4^. To dynamically adapt the learning rate during training, we used a cosine decay learning rate scheduler, optimizing the convergence and fine-tuning process. Furthermore, to enhance the training efficiency and minimize memory consumption, we used float16 precision, a half-precision training technique, which effectively balances training speed and model performance. This comprehensive approach allowed us to train our model effectively, incorporating LoRA’s enhancements for improved performance and robustness. All the experiments are conducted on a graphics processing unit (NVIDIA V100).

To evaluate the performance of the ClinicalBLIP model, we compared our method with the following 11 state-of-the-art methods. R2GEN [9] is a memory-driven radiology report generation model with a relational memory to record the information from the previous generation processes and a layer normalization mechanism to incorporate the memory. CA [26] is a contrastive attention model to capture and depict abnormalities by comparing the input image with known normal images. CMCL [27] is a novel competence-based multimodal curriculum learning framework to alleviate data bias by efﬁciently using limited medical data for medical report generation. Posterior-and-Prior Knowledge Exploring-and-distilling [28] is an effective approach to exploring and distilling posterior and prior knowledge for radiology report generation. R2GEN enhanced with cross-modal memory networks [29] is a radiology report generation model with cross-modal memory networks in which a memory matrix is used to record the alignment and interaction between images and texts, and another memory is used to perform querying and responding to obtain the shared information across modalities. ALIGNTRANSFORMER [30] is a radiology report generation model to alleviate the data bias problem and model the very long sequence. Knowledge Matters [31] is a novel radiology generation framework assisted by general and speciﬁc knowledge. Meshed-Memory Transformer [32] is a simple but effective progressive text generation model to produce the radiology report by incorporating high-level concepts into the generation progress. Reinforcement Learning Over a Cross-Modal Memory (CMM-RL) [33] is an enhanced radiology report generation model with reinforced cross-modal alignment to alleviate the requirement of annotated supervision while facilitating interactions across modalities. Cross-Modal Contrastive Attention (CMCA) [34] is a novel model to capture both visual and semantic information from similar cases. Observation-Guided Radiology Report Generation (ORGAN) [35] is a generation framework that can maintain the clinical consistency between radiographs and generated free-text reports.

We adopted natural language generation metrics to evaluate the methods. Specifically, we selected Bilingual Evaluation Understudy (BLEU) [36], Metric For Evaluation of Translation with Explicit Ordering (METEOR) [37], and Recall-Oriented Understudy for Gisting Evaluation (ROUGE) [38]. BLEU-1, BLEU-2, BLEU-3, BLEU-4, METEOR, and ROUGE-L are reported.

BLEU is primarily used to evaluate the quality of machine-generated translations by comparing them to 1 or more reference translations. It computes a precision-based metric by counting the number of n-grams (contiguous sequences of n items, usually words) in the generated translation that matches any reference translation. In this work, BLEU is used to evaluate the generated text report.

METEOR is based on the harmonic mean of unigram precision and recall, with recall weighted higher than precision. It incorporates features not found in other metrics, such as stemming and synonymy matching, along with standard exact word matching. The metric was designed to address some of the issues found in the more popular BLEU metric and to produce a good correlation with human judgment at the sentence or segment level.

ROUGE compares an automatically produced text against a reference or a set of reference text. It measures the overlap of n-grams and word sequences between the generated text and reference text. ROUGE captures both precision and recall, providing a more balanced evaluation, and can be adapted for different summary lengths.

### Ethical Considerations

This study complied with all relevant ethical regulations. All the publicly available data sets have been deidentified and anonymized. With institutional review board approval (OHSRP#5357) by the National Institutes of Health Office of Human Research Protection Programs, the IU X-RAY data set was made publicly available by Indiana University, and no informed consent was necessary [1]. The MIMIC-CXR data set was originally approved by the institutional review board of the Beth Israel Deaconess Medical Center and the requirement for individual patient consent was waived [20].

## Results

### Quantitative Evaluation

Tables 1 and 2 provide the quantitative results of the IU X-RAY and MIMIC-CXR test sets, respectively. The detailed results show that the ClinicalBLIP model exhibited robust performance when compared with other methods across the IU X-RAY and MIMIC-CXR data sets. For the IU X-RAY data set, as shown in Table 1, although ClinicalBLIP was slightly inferior to the competitor methods on some individual metrics, it significantly surpassed the competitor methods on most metrics. With a BLEU-A score of 0.296, it boasted an improvement of roughly 6.9% over its nearest competitor, CMCA, which had a BLEU-A score of 0.277. This showcases ClinicalBLIP’s enhanced capability in producing reports that are more aligned with the reference. Moreover, when assessing the METEOR metric, which provides insights into the robustness of generation, ClinicalBLIP achieved a score of 0.570. This was approximately 1.7 times higher than CMCA’s 0.209, reflecting ClinicalBLIP’s superior relevance to the generated report. The ROUGE-L metric further solidified this observation; ClinicalBLIP’s score of 0.534 was about 33.8% higher than ORGAN’s score of 0.399, suggesting that ClinicalBLIP consistently maintained a high level of linguistic quality and relevance in its results.

For the MIMIC-CXR data set, as shown in Table 2, there were areas where ClinicalBLIP did not have the highest score, but its comprehensive performance remains commendable. The BLEU-A score for ClinicalBLIP stood at 0.162, which, while marginally behind ORGAN’s score of 0.184, indicates a competitive translation quality. However, ClinicalBLIP made a strong comeback in the METEOR metric, recording a score of 0.365, which is approximately 1.25 times higher than ORGAN’s score of 0.162. This underlines ClinicalBLIP’s proficiency in generating semantically relevant reports. Furthermore, with a ROUGE-L score of 0.313, ClinicalBLIP managed to surpass ORGAN by roughly 6.8%, emphasizing its consistent linguistic excellence.

In summary, while individual metrics might have seen close competition, the overall trend clearly indicates the comprehensive strength of the ClinicalBLIP model. Its consistently high scores across various data sets and metrics demonstrate its versatility and reliability in the realm of clinical report generation.

**Table 1:** The BLEU^a^, METEOR^b^, and ROUGE-L^c^ scores of the generated reports by various methods on the IU X-RAY data set.

Methods	IU X-RAY						
	BLEU-1	BLEU-2	BLEU-3	BLEU-4	BLEU-A^d^	METEOR	ROUGE-L
R2GEN	0.470	0.304	0.219	0.165	0.229	N/A^e^	0.371
CA^f^	0.492	0.314	0.222	0.169	0.235	0.193	0.381
CMCL^g^	0.473	0.305	0.217	0.162	0.228	0.186	0.378
PPKED^h^	0.483	0.315	0.224	0.168	0.236	N/A	0.376
M2TR^i^	0.475	0.309	0.222	0.170	0.234	0.191	0.375
R2GENCMN^j^	0.486	0.317	0.232	0.173	0.241	0.192	0.390
ALIGNTRANSFORMER	0.484	0.313	0.225	0.173	0.237	N/A	0.379
KNOWMAT^k^	0.496	0.327	0.238	0.178	0.248	N/A	0.381
CMM-RL	0.494	0.321	0.235	0.181	0.246	0.201	0.384
CMCA^l^	0.496	0.349	0.268	0.215	0.277	0.209	0.392
ORGAN^m^	0.510	0.346	0.255	0.195	0.265	0.205	0.399
ClinicalBLIP	0.433	0.343	0.290	0.254	0.296	0.570	0.534

^a^BLEU: Bilingual Evaluation Understudy.

^b^METEOR: Metric for Evaluation of Translation With Explicit Ordering.

^c^ROUGE-L: Recall-Oriented Understudy for Gisting Evaluation-L.

^d^BLEU-A: average of the BLEU-2/3/4 scores.

^e^N/A: not available.

^f^CA: contrastive attention.

^g^CMCL: competence-based multimodal curriculum learning.

^h^PPKED: Posterior-and-Prior Knowledge Exploring-and-distilling.

^i^M2TR: Meshed-Memory Transformer.

^j^R2GENCMN: R2GEN enhanced with cross-modal memory networks.

^k^KNOWMAT: Knowledge Matters.

^l^CMCA: Cross-Modal Contrastive Attention Model.

^m^ORGAN: Observation-Guided Radiology Report Generation Framework.

**Table 2:** The BLEU^a^, METEOR^b^, and ROUGE-L^c^ scores of the generated reports by various methods on the MIMIC-CXR data set.

Methods	MIMIC-CXR						
	BLEU-1	BLEU-2	BLEU-3	BLEU-4	BLEU-A^d^	METEOR	ROUGE-L
R2GEN	0.353	0.218	0.145	0.103	0.155	0.142	0.270
CA^e^	0.350	0.219	0.152	0.109	0.160	0.151	0.283
CMCL^f^	0.344	0.217	0.140	0.097	0.151	0.133	0.281
PPKED^g^	0.360	0.224	0.149	0.106	0.160	0.149	0.284
M2TR^h^	0.353	0.218	0.148	0.106	0.157	0.142	0.278
R2GENCMN^i^	0.378	0.232	0.154	0.107	0.164	0.145	0.272
ALIGNTRANSFORMER	0.378	0.235	0.156	0.112	0.168	N/A^j^	0.283
KNOWMAT^k^	0.363	0.228	0.156	0.115	0.166	N/A	0.284
CMM-RL	0.381	0.232	0.155	0.109	0.165	0.151	0.287
CMCA^l^	0.360	0.227	0.156	0.117	0.167	0.148	0.287
ORGAN^m^	0.407	0.256	0.172	0.123	0.184	0.162	0.293
ClinicalBLIP	0.332	0.219	0.153	0.115	0.162	0.365	0.313

^a^BLEU: Bilingual Evaluation Understudy.

^b^METEOR: Metric for Evaluation of Translation With Explicit Ordering.

^c^ROUGE-L: Recall-Oriented Understudy for Gisting Evaluation-L.

^d^BLEU-A: average of the BLEU-2/3/4 scores.

^e^CA: contrastive attention.

^f^CMCL: Competence-Based Multimodal Curriculum Learning.

^g^PPKED: Posterior-and-Prior Knowledge Exploring-and-distilling.

^h^M2TR: Meshed-Memory Transformer.

^i^R2GENCMN: R2GEN enhanced with cross-modal memory networks.

^j^N/A: not available.

^k^KNOWMAT: Knowledge Matters.

^l^CMCA: Cross-Modal Contrastive Attention Model.

^m^ORGAN: Observation-Guided Radiology Report Generation Framework.

### Ablation Study

We also conducted an ablation study to analyze the impact of fine-tuning on different modules, such as the original InstructBLIP (without any fine-tuning on this task), LLM, visual encoder, and prior information, and show the results in Table 2. Based on the ablation study results presented in Table 3, several observations can be made regarding the performance of different methods on the IU X-RAY data set. The ClinicalBLIP method achieved a BLEU score of 0.296, a METEOR score of 0.570, and a ROUGE-L score of 0.534, indicating its robust performance across the metrics. When the effective tuning was removed, namely InstructBLIP, there was a significant drop in all metrics, especially in the BLEU score, which dropped to a mere 0.011. This highlights the importance of effective tuning for the model’s performance. Similarly, removing prior information also led to a decline in performance, with the METEOR metric showing a noticeable drop, to 0.339. The removal of LLM tuning and visual encoder tuning resulted in reduced scores, but this was not as drastic as in the former cases. The BLEU score dropped to 0.149 and 0.245, respectively, while the METEOR score was 0.458 and 0.513 for the same conditions.

In summary, effective fine-tuning and prior information played a vital role in achieving optimal performance, and LLM tuning and visual encoder tuning were also important components for enhancing the model’s results. All the components together contributed to the best results.

**Table 3:** Experimental results of ablation study on the IU X-RAY test set.

Methods	BLEU-A^a^ score	METEOR^b^ score	ROUGE-L^c^ score
ClinicalBLIP with all fine-tuning	0.296	0.570	0.534
ClinicalBLIP without effective tuning	0.011	0.096	0.057
ClinicalBLIP without prior information	0.091	0.339	0.283
ClinicalBLIP without LLM^d^ tuning	0.149	0.458	0.412
ClinicalBLIP without visual encoder tuning	0.245	0.513	0.474

^a^BLEU-A: the average of the BLEU-2/3/4 scores.

^b^METEOR: Metric for Evaluation of Translation With Explicit Ordering.

^c^ROUGE-L: Recall-Oriented Understudy for Gisting Evaluation–L.

^d^LLM: large language model.

## Discussion

### Principal Results

Our proposed model, ClinicalBLIP, achieved the best METEOR and ROUGE-L scores and competitive BLEU scores on the test sets of both IU X-RAY and MIMIC-CXR. The primary outcomes of this study are to (1) propose a multistage fine-tuning strategy that separately enhances the visual encoder and the LLM’s understanding of medical image and text, allowing the LLM to harness the knowledge acquired during the pretraining process and (2) incorporate the medical tags of medical images and brief introductions of these images in the form of prompts into the model’s training and prediction processes, the large model can effectively combine the introduced text-based prior knowledge with medical images to generate a more accurate report. Experimental results demonstrate that ClinicalBLIP has great potential to help medical experts facilitate radiology report generation and improve the efficiency of decision-making for clinical diagnosis and treatment.

### Case Study

**Figure 5:**
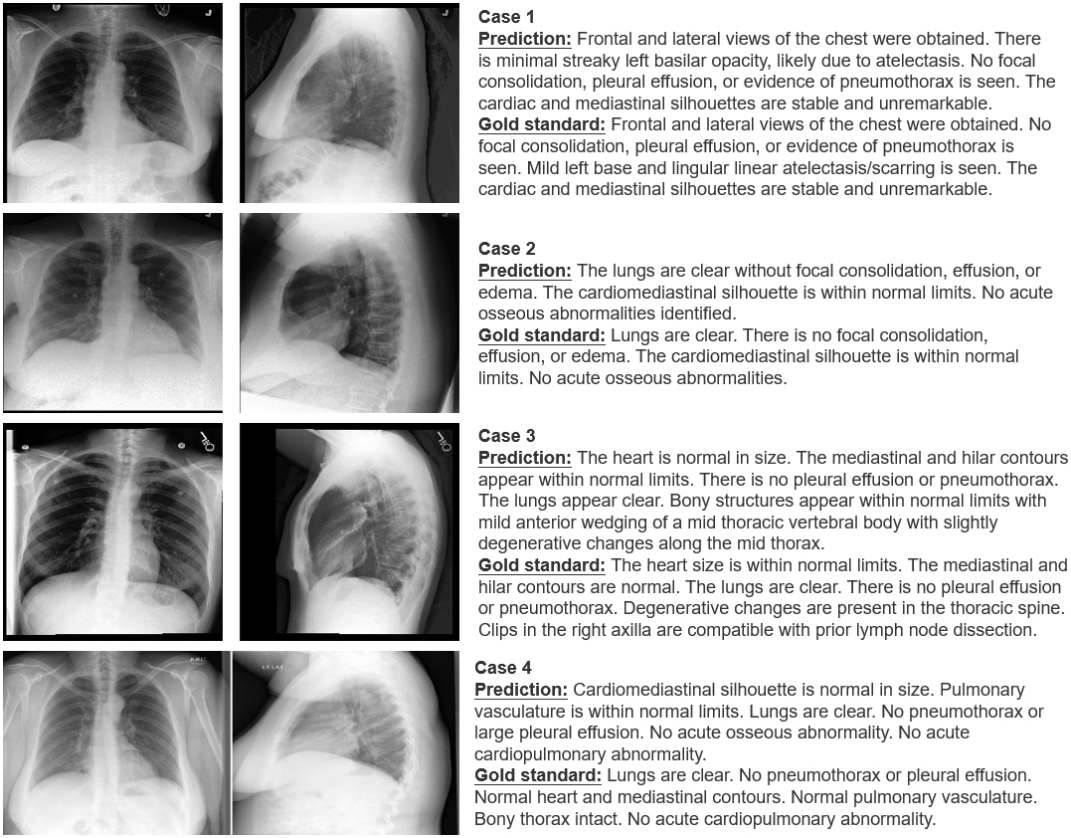
Cases generated by ClinicalBLIP on the MIMIC-CXR test set.

In addition to quantitative evaluations, we conducted an extensive set of qualitative case studies to analyze the generated report. Figure 5 shows 4 cases selected from the generated reports on the MIMIC-CXR test set.

By comparing the prediction and the gold standard, it can be found that case 1 and case 2 are good cases. For case 1, although the prediction and the gold standard are not exactly the same, there are differences in the order of symptom descriptions and word choices; the deep semantic meanings expressed by the two are basically consistent. However, the gold standard provides more details than the prediction, which also explains why the BLEU score is not ideal in certain situations. For case 2, both the prediction and the gold standard reports are closely aligned and convey the same overall findings. The patient’s chest x-ray does not reveal any significant abnormalities. This is a good case as it highlights the consistency and accuracy of radiological interpretation.

Besides the first 2 good cases, there are also areas that need improvement and enhancement. Cases 3 and 4 in Figure 5 show 2 bad cases. For case 3, both the prediction and the gold standard state that the heart is within the normal size, and the lungs appear clear with no signs of pleural effusion or pneumothorax. However, the prediction mentions mild anterior wedging of a midthoracic vertebral body with slight degenerative changes along the midthorax. In contrast, the the gold standard report mentions degenerative changes in the thoracic spine but does not specify the location or type of degeneration. The discrepancies in the description of the bony structures between the prediction and the the gold standard report could also be of concern. Different types and locations of degenerative changes can have different clinical implications. For case 4, while the prediction and the gold standard largely align on most observations, there are subtle differences in phrasing. For instance, the prediction mentions the cardiomediastinal silhouette is normal in size, whereas the the gold standard emphasizes the normal contours of the heart and mediastinum. Such subtle linguistic variations can potentially lead to misunderstandings in diagnosis or interpretation, especially in critical medical decisions. Therefore, even though the general assessments align, precision in wording remains essential.

In summary, it is crucial to ensure that automatic or artificial intelligence–based predictions in radiology are meticulously validated and cross-referenced with expert opinions to ensure patient safety and accurate diagnosis.

### Comparison With Prior Work

In the medical or clinical field, there has been a surging interest in developing artificial intelligence applications for image captioning, that is, radiology report generation. Most studies have focused on improving the quality of the generated report by using cross-modal memory to facilitate the generation process [28], reinforcing learning to align the cross-modal information [32], and planning and iterative refinement for long text generation [25]. However, these methods have not explored the capabilities of large VLMs for this task. In this study, we successfully applied large VLMs to the radiology report generation task by designing effective multistage fine-tuning strategies and incorporating prior knowledge mechanisms. We validated our approach on multiple task data sets and achieved state-of-the-art performance.

### Limitations and Future Work

Although ClinicalBLIP has made significant strides and shown promising outcomes, there are still some unresolved issues. As mentioned above, ClinicalBLIP has discrepancies in terminological expressions in some cases compared to the the gold standard and sometimes lacks or misinterprets comprehensive details in certain descriptions. Therefore, in future work, we will continue to optimize ClinicalBLIP, considering the integration of reasoning techniques like chain of thoughts into the fine-tuning process. This aims to enhance the model’s semantic consistency in professional expressions and provide more detailed descriptions while also verifying the model’s generalization capabilities on more data sets. Moreover, we will seek collaboration from professional practitioners, including both directions for model improvement and methods for model evaluation.

### Conclusions

In this study, the ClinicalBLIP model was introduced, leveraging large VLMs for radiology report generation. Tested on the IU X-RAY/MIMIC-CXR data sets, ClinicalBLIP significantly outperformed several competitor methods in METEOR and ROUGE scores, showcasing its potential to enhance automatic report generation in clinical radiology and streamline patient care processes.

## Abbreviations

BERT: bidirectional encoding representation of transformer
BLEU: Bilingual Evaluation Understudy
CMCA: Cross-Modal Contrastive Attention
CMM-RL: Reinforcement Learning Over a Cross-Modal Memory
CNN: convolutional neural network
LLM: large language model
LoRA: low-rank adaptation
METEOR: Metric for Evaluation of Translation With Explicit Ordering
MLP: multilayer perceptron
ORGAN: Observation-Guided Radiology Report Generation
Q-Former: query transformer
RNN: recurrent neural network
ROUGE: Recall-Oriented Understudy for Gisting Evaluation
VLM: vision-language model
